# Minimal residual disease negativity by next-generation flow cytometry is associated with improved organ response in AL amyloidosis

**DOI:** 10.1038/s41408-021-00428-0

**Published:** 2021-02-16

**Authors:** Giovanni Palladini, Bruno Paiva, Ashutosh Wechalekar, Margherita Massa, Paolo Milani, Marta Lasa, Sriram Ravichandran, Isabel Krsnik, Marco Basset, Leire Burgos, Mario Nuvolone, Ramón Lecumberri, Andrea Foli, Noemi Puig, Melania Antonietta Sesta, Margherita Bozzola, Pasquale Cascino, Alice Nevone, Jessica Ripepi, Pierpaolo Berti, Simona Casarini, Ombretta Annibali, Alberto Orfao, Jesus San-Miguel, Giampaolo Merlini

**Affiliations:** 1 Amyloidosis Research and Treatment Center, “Fondazione Istituto di Ricovero e Cura a Carattere Scientifico (IRCCS) Policlinico San Matteo”, Pavia, Italy; 2 Biochemistry, Biotechnology and Advanced Diagnostics Laboratory, “Fondazione Istituto di Ricovero e Cura a Carattere Scientifico (IRCCS) Policlinico San Matteo”, Pavia, Italy; 3 Department of Molecular Medicine, University of Pavia, Pavia, Italy; 4 Clinica Universidad de Navarra, Centro de Investigacion Medica Aplicada (CIMA), IDISNA, CIBERONC CB16/12/00369 Pamplona, Pamplona, Spain; 5 National Amyloidosis Centre, University College London (Royal Free Campus), London, UK; 6 Department of Haematology, University College London Hospitals, London, UK; 7 Hospital Universitario Puerta del Hierro, Madrid, Spain; 8 Servicio General de Citometría, Universidad de Salamanca, IBSAL, and IBMCC CSIC-USAL, CIBERONC, Salamanca, Spain; 9 Immunohematology and Transfusion Medicine Unit, Department of Laboratories, IRCCS Bambino Gesù Children’s Hospital, Rome, Italy; 10 Hematology and Stem Cell Transplantation Unit, University Campus Bio-medico, Rome, Italy

**Keywords:** Translational research, Disease-free survival

## Abstract

Light chain (AL) amyloidosis is caused by a small B-cell clone producing light chains that form amyloid deposits and cause organ dysfunction. Chemotherapy aims at suppressing the production of the toxic light chain (LC) and restore organ function. However, even complete hematologic response (CR), defined as negative serum and urine immunofixation and normalized free LC ratio, does not always translate into organ response. Next-generation flow (NGF) cytometry is used to detect minimal residual disease (MRD) in multiple myeloma. We evaluated MRD by NGF in 92 AL amyloidosis patients in CR. Fifty-four percent had persistent MRD (median 0.03% abnormal plasma cells). There were no differences in baseline clinical variables in patients with or without detectable MRD. Undetectable MRD was associated with higher rates of renal (90% vs 62%, *p* = 0.006) and cardiac response (95% vs 75%, *p* = 0.023). Hematologic progression was more frequent in MRD positive (0 vs 25% at 1 year, *p* = 0.001). Altogether, NGF can detect MRD in approximately half the AL amyloidosis patients in CR, and persistent MRD can explain persistent organ dysfunction. Thus, this study supports testing MRD in CR patients, especially if not accompanied by organ response. In case MRD persists, further treatment could be considered, carefully balancing residual organ damage, patient frailty, and possible toxicity.

## Introduction

Light chain (AL) amyloidosis is caused by a small B-cell clone, more commonly a plasma cell (PC) clone with shared genetic features with multiple myeloma (MM) and monoclonal gammopathy of undetermined significance, producing light chains (LCs) that form amyloid deposits and exert toxicity on target organs^1–3^. Lymphoplasmacytic clones sustain the disease in a minority of patients^4^. The size of the underlying PC clone at baseline affects prognosis^5^. Profound decreases of LC levels through PC-targeting chemotherapy can result in the improved organ dysfunction and arrest the otherwise inexorable progression of the disease^6–10^. After treatment, even small increases in amyloid LCs can cause organ progression and reduced survival^11^. Current hematologic and organ response criteria are based on M protein studies and on changes in the difference between amyloidogenic (involved; iFLC) and uninvolved free LC (dFLC) and in markers of organ dysfunction^12,13^. Amyloid complete response (aCR) is defined by normal FLC ratio plus negative serum and urine immunofixation, and predicts prolonged survival^12^. However, even aCR does not translate into organ response in all patients. This could be explained by irreversible organ damage, permanence of amyloid deposits, or persistence of undetectable levels of amyloid LCs produced by treatment-resistant residual PCs, possibly combined.

High-sensitive next-generation flow cytometry (NGF) is used to detect minimal residual disease (MRD) in MM^14^. As compared to previous, less sensitive flow cytometry methods, NGF offers robust surrogate endpoints for clinical trials and guidance for treatment^14–18^. Accordingly, it has been suggested as the new treatment endpoint for MM^19^. Evaluation of MRD in AL amyloidosis is an emerging area of interest^20–27^. The group at Mayo Clinic showed that lack of clonal bone marrow (BM) PCs by standard-sensitivity multiparameter flow cytometry, is associated with improved progression-free survival^25,26^. More recently, the Boston group reported trend for higher probability of organ response in patients in aCR and undetectable MRD^28^. However, there are no studies evaluating the incremental clinical significance of MRD monitoring by high-sensitivity NGF over routine monoclonal protein studies for hematologic response assessment in patients with AL amyloidosis otherwise in aCR. Thus, it remains unknown if, similarly to MM^16^, a flow MRD-negative criteria should be adopted in AL amyloidosis.

## Methods

From April 2016 to July 2019, 92 patients confirmed to be in aCR at least 6 months after treatment discontinuation were selected for NGF-based MRD assessment at 14 participating centers in Italy, Spain, and the United Kingdom (Supplemental Table 1). Patients with MM, defined as >60% BMPC, and/or involved/uninvolved FLC ratio >100, and/or bone lesions, as well as patients with IgM-related AL amyloidosis were excluded. All patients gave written informed consent for their clinical data to be used for research purposes.

Clinical variables were recorded at the time of diagnosis, at the time of first documentation of aCR, at the time of MRD assessment, and at each subsequent evaluation. Clinical decisions (e.g., treatment and frequency of subsequent evaluations) were protocolized and were not influenced by the presence or absence of MRD, except for a single patient with MRD and organ progression who started rescue treatment. All patients who maintained aCR 6 months after the first assessment were asked to perform BM aspiration for MRD evaluation. The patients who no longer satisfied criteria of aCR were excluded from the final analysis.

Hematologic and organ responses and hematologic relapse from aCR were defined based on current criteria^12,13,29^. aCR required both negative serum and urine immunofixation and normal FLC ratio.

Cardiac and renal responses were evaluated at the time of first documentation of aCR (compared to data at diagnosis) and at the time of MRD assessment (compared to data at the time of first documentation of aCR). Organ response was also assessed comparing data obtained at the time of MRD assessment with those obtained at the time of diagnosis. Cardiac response was defined as a decrease both >30% and >300 ng/L in NT-proBNP. The NT-proBNP level needed to be >650 ng/L for cardiac response to be measurable. Renal response was defined as a decrease of at least 30% or <0.5 g/24 h of proteinuria in the absence of a decrease >25% in estimated glomerular filtration rate in patients whose proteinuria was at least 0.5 g/24 h.

Hematologic relapse from aCR was defined by the reappearance of a monoclonal component in serum and or urine at immunofixation, and/or by an abnormal FLC ratio.

NGF cytometry on BM aspirates was performed according to the EuroFlow protocol at two different centers (Supplemental Table 1), with harmonized pre-analytical and analytical procedures. Samples not processed and analyzed locally were shipped overnight to one of the two evaluating centers (Supplemental Table 1). NGF-based antibody combinations were used for characterizing MRD in BM aspirates (according to EuroFlow)^14^. Briefly, the EuroFlow lyse-wash-and-stain standard sample preparation protocol and the 2-tube 8-color EuroFlow NGF antibody panel was performed for identification of BMPC, and discrimination between phenotypically aberrant and normal PC. Tube 1 included CD138-BV421, CD27-BV510, CD38-FITC, CD56-PE, CD45-PerCPCy5.5, CD19-PECy7, CD117-APC, and CD81-APCH7 monoclonal antibodies. In tube 2, the CD117-APC and CD81-APCH7 monoclonal antibodies were replaced by CyIgKappa-APC and CyIgLambda-APCH7, respectively. Labeled antibodies were purchased from Cytognos S.L. (Salamanca, Spain), BD Biosciences (San Jose, CA, USA), BioLegend Inc. (San Diego, CA, USA), Beckman Coulter (Brea, CA, USA), and DAKO (Glostrup, Denmark). The two-tube strategy allows detection of clonality with specific confirmation of LC restriction on phenotypically aberrant PC, identified by antigen under-expression (CD19, CD27, CD38, CD45, and CD81) or overexpression (CD56, CD117, CD138) as compared to normal PCs. In accordance with the guidelines for MRD response criteria in MM^16^, a minimum sensitivity of 10^−5^ was achieved in all patients and 10^−6^ sensitivity was reached in 70/92 (76%) cases. Data were analyzed using the Infinicyt software (version 1.7; Cytognos Salamanca, Spain) by operators blind to clinical data. The percentage of B-cell precursors, nucleated red blood cells, and mast cells was evaluated in each sample to determine the extent of hemodilution.

Fisher exact test was used to assess differences between subgroups, and long-rank test to compare times to progression.

## Results

A total of 108 patients with AL amyloidosis who were known to be in aCR at least 6 months after treatment discontinuation, underwent NGF-based MRD assessment. Sixteen patients (15%) did not maintain aCR at the time the BM specimen for MRD assessment was obtained, and were excluded for the study. In these 16 subjects, a median of 447 PCs with abnormal phenotype (range 54–3581), corresponding to 0.02% (range 0.002–0.335%) were detected. The remaining, 92 patients with confirmed aCR at the time of MRD assessment were considered for the analysis (Table 1). Median time between first achievement of aCR and MRD assessment was 11 months (interquartile range 9–30 months). Patients were exposed to one (52 cases, 56%) or two (40 cases, 44%) lines of therapy before aCR was reached and MRD was assessed.

**Table 1:** Patients characteristics.

Variable	MRD positive (*N* = 50) *N* (%)/median (IQR)	MRD negative (*N* = 42) *N* (%)/median (IQR)	*P* value
Male sex	33 (66)	21 (50)	0.128
Age at diagnosis, years	59 (55–66)	61 (55–68)	0.280
Organ involvement at diagnosis			
Heart	36 (67)	23 (54)	0.093
Kidney	30 (60)	31 (73)	0.172
Liver	11 (22)	4 (9)	0.117
Cardiac stage at diagnosis	(Available in 45 patients)	(Available in 40 patients)	
I	11 (24)	17 (43)	0.084
II	18 (40)	12 (30)	0.341
IIIa	15 (34)	9 (22)	0.227
IIIb	1 (2)	2 (5)	0.582
Renal stage at diagnosis	(Available in 41 patients)	(Available in 39 patients)	
I	20 (49)	18 (46)	0.818
II	18 (44)	18 (46)	0.843
III	3 (7)	3 (8)	0.951
eGFR, mL/min per 1.73 m^2^	86 (61–90)	76 (60–89)	0.158
BMPC at diagnosis (%)	8 (4–14)	9 (4–15)	0.865
dFLC at diagnosis, mg/L	141 (65–-488)	112 (34–397)	0.303
Exposure to two lines of therapy before aCR	25 (50)	15 (36)	0.177
Time from diagnosis to first aCR documentation, months	10 (6–15)	11 (5–19)	0.823
Time from first aCR documentation to MRD assessment, months	13 (5–30)	12 (6–37)	0.368
Autologous stem cell transplant	19 (38)	16 (38)	0.991
Melphalan	23 (46)	14 (33)	0.227
Bortezomib	45 (90)	36 (86)	0.547
Cardiac response at the time aCR was documented	16/29 (55)	15/21 (71)	0.262
Renal response at the time aCR was documented	12/29 (41)	19/31 (61)	0.470

Cardiac stage is defined by N-terminal pro-natriuretic peptide type B (NT-proBNP, cutoff 332 ng/L) and cTnI (cutoff 0.1 ng/mL), with stages I–III patients having none, one, or to markers above the cutoff, respectively. Stage IIIa patients have NT-proBNP <8500 ng/L. Stage IIIb patients have NT-proBNP >8500 ng/L. Renal stage is defined by eGFR (cutoff 50 mL/min per 1.73 m^2^) and proteinuria (cutoff 5 g/24 h); stage I patients have both eGFR above and proteinuria below the cutoff, stage II have either eGFR below or proteinuria above the cutoff, and stage III patients have both eGFR below and proteinuria above the cutoff.

*eGFR* estimated glomerular filtration rate, *dFLC* difference between involved (amyloidogenic) and uninvolved free light chain, *aCR* amyloid complete response defined by negative serum and urine immunofixation and normal free light chain ratio, BMPC bone marrow plasma cell infiltrate, MRD minimal residual disease.

Fifty patients (54%) had detectable MRD (median 0.02701%, range 0.0002–0.33010%). There was no significant difference in clinical variables measured at baseline in patients with and without detectable MRD. Moreover, the number of lines of treatment performed before evaluation, the median time from diagnosis to first aCR documentation and the median time from first aCR documentation to MRD assessment were not different between MRD positive and negative patients. At the time of MRD assessment, dFLC levels ranged from 0 mg/L to 29 mg/L and were <10 mg/L in 67 patients (73%) in the entire study population. Of note, dFLC levels were significantly lower in patients with undetectable MRD (median 1.5 vs 6.5 mg/L, *P* = 0.001).

Patients with undetectable MRD were more likely to attain renal [92% (23/25 evaluable) vs 57% (15/26 evaluable), *P* = 0.005] and cardiac response [95% (18/19 evaluable) vs 71% (20/28 evaluable), *P* = 0.046]. Importantly, time between diagnosis and aCR, or between achievement of aCR and MRD assessment was not associated with organ response (Supplementary Table 2). As above mentioned, organ response was assessed by comparing the organ function at the time of MRD assessment with data obtained at the time of diagnosis. Interestingly, the rate of renal response was higher in the MRD negative cohort: 90% (28/31 evaluable) vs 62% (18/29 evaluable), *P* = 0.006]. The same pattern was observed for cardiac response: 95% (22/23 evaluable) vs 75% (24/32 evaluable), *P* = 0.023] for patients with undetectable vs detectable MRD, respectively.

**Fig. 1:**
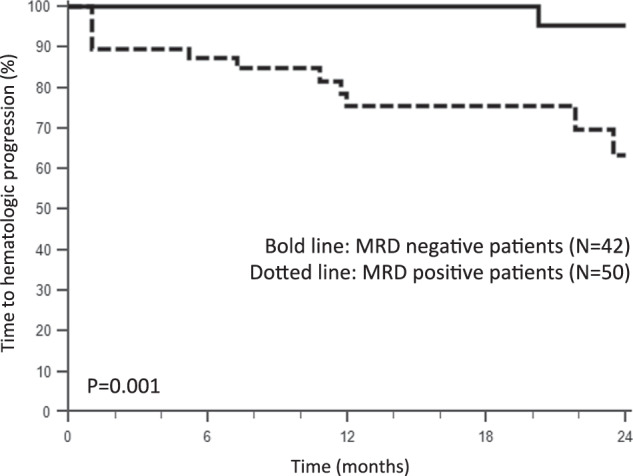
Maintenance of amyloid complete response after MRD assessment according to MRD status. Bold line: MRD negative patients (*N* = 42). Dotted line: MRD positve patients (*N* = 50).

After a median follow-up of 23 months from the time of MRD assessment, three patients, all with persistent MRD, died. The difference in overall survival between patients with and without detectable MRD did not reach statistical significance (*P* = 0.203). Time to hematologic progression was significantly longer in MRD-negative patients: only 1 patient with undetectable MRD (sensitivity reached, 10^−5^) progressed, compared to 13 MRD-positive patients (Fig. 1). Interestingly, rate of hematologic progression at 1 year after MRD assessment was not different in patients who received one or two lines of therapy (10% vs 15% progressing at 1 year, *P* = 0.763) and in patients who received or did not receive autologous stem cell transplant (5% vs 10% progressing at 1 year, *P* = 0.278).

## Discussion

We report here on the largest population of patients with AL amyloidosis with sustained CR undergoing MRD assessment with NGF. With a minimum sensitivity of 10^−5^ (reaching 10^−6^ in 76% of cases), persistent MRD was identified in 54% of cases. This proportion does not appear to be affected by exposure to one vs two lines of therapy, and time to achievement of aCR or to assessment of MRD. This finding is in agreement with the observations by Kastritis et al. who analyzed 20 patients in aCR (40% after ASCT) and reported MRD negativity in 8 (40%)^24^. Similarly, Muchtar et al. analyzed patients at the end of first-line treatment (84% after ASCT) and reported that, among 16 subjects in aCR, 8 (50%) had undetectable clonal PCs by multiparameter flow cytometry^26^. In a more recent case series from the Mayo Clinic, where MRD assessment was performed within 2 years from start of therapy (in 57% of cases after ASCT), MRD negativity was observed in 15 out of 20 (75%) patients in aCR. In the Boston series, 55% of patients in CR were MRD positive and a trend to a better organ (especially renal) response was noted in those who reached MRD negativity. While small sample size and differences in patient selection criteria may at least partially account for the increased rate of MRD negativity in this study, the lower sensitivity of MRD assessment (≥10^−5^, with 11 out of 22 patients in VGPR found to be MRD negative) could also have played a role. Table 2 summarizes the methodology and findings of published flow cytometry-based MRD studies in AL amyloidosis and of the present report. The fact that currently available therapies can yield profound, MRD-negative responses in up to 50% of patients with AL amyloidosis in aCR is encouraging and can explain the long-term progression-free survival of patients in aCR. These promising results will probably improve when novel, powerful drugs such as daratumumab, will become accessible.

**Table 2:** Methodology and findings of published flow cytometry-based MRD studies in AL amyloidosis and of the present report.

Study	NGF technique	Sensitivity	*N*. pts	Pts. in CR *N* (%)	ASCT *N* (%)	Eligibility criteria	Median time from CR to MRD assessment	MRDpos *N* (%)	Median follow-up from MRD assessment	% Hematologic progression POS vs NEG	Cardiac response *N*/evaluable (%) POS vs NEG	Renal response *N*/evaluable (%) POS vs NEG
Kastritis et al.^24^	EuroFlow	Range: 2–3.1 × 10^−6^ Median 2.3 × 10^−6^	20	20 (100%)	8 (40%)	CR+ negative BM biopsy	36 months	12 (60%)	NA	NA	1/4(25%) vs 3/3(100%)	6/10(60%) vs 6/8 (75%)
Muchtar et al.^26^	7-color MFC	Range: 1 × 10^−4^–2 × 10^−5^	82	16 (20%)	69 (84%)	End of first-line treatment	NA	58 (71%)^a^ Subgroup of CR pts.: 8 (50%)	NA	3-year PFS: 28% vs 88% Subgroup of CR pts.: 33% vs 100%	Subgroup of VGPR/CR pts.: 10/12 (83%) vs 8/8 (100%)	Subgroup of VGPR/CR pts.: 13/19 (68%) vs 14/14 (100%)
Sidana et al.^25^	Euroflow	Range: ≥1 × 10^−5^–10^−6^	44	20 (45%)	25 (57%)	MRD testing within 2 years from start of therapy	NA	14 (36%)^b^ Subgroup of CR pts.: 5 (25%)	14 months	Estimated 1-year PFS: 64% vs 100%	2/7 (22%) vs 8/12 (67%)	8/9 (89%) vs 11/16 (69%)
Staron et al.^28^	2-tube, 10-color antibody panel	Range: 1 × 10^−4^–1 × 10^−5^	65	65 (100%)	32 (49%)	CR at previous evaluation	71 months for MRD negative 32 months for MRD positive	36 (55%)	NA	NA	10/17 (59%) vs 9/12 (75%)	18/28 (64%) vs 21/24 (88%)
Kastritis et al.^27^	Euroflow	Range: 2 × 10^−6^–3.1 × 10^−6^	52	52 (100%)	7 (14%)	CR after their primary therapy	6 months (range 3–12)	28 (55%)	24 months	21% vs 0%	11/15 (73%) vs 10/10 (100%)	14/16 (87.5%) vs 15/17 (88%)
Current study	Euroflow	10^−6^: 76% 10^−5^: 24%	92	92 (100%)	35 (38%)	CR confirmed at least 6 months after end of therapy	MRD pos: 13 months; MRD neg: 12 months	50 (54%)	23 months	26% vs 2%	18/19 (95%) vs 20/28 (71%)	23/25 (92%) vs 15/26 (57%)

*ASCT* autologous stem cell transplantation, *BM* bone marrow, *CR* complete response (hematologic), *MFC* multiparametric flow cytometry, *MRD* minimal residual disease, *NA* not available, *NGF* next-generation flow, *NEG* negative, *POS* positive, *PFS* progression-free survival, *pts*. patients, *VGPR* very good partial response.

^a^MRD negativity in 16 out of 22 patients in VGPR.

^b^MRD negativity in 2 patients with less than VGPR and in 11 out of 22 patients in VGPR.

In the present study, undetectable MRD was associated with a further improvement of organ involvement after aCR in >90% of patients. Both renal and cardiac response rates were higher in the MRD-negative cohort and also from diagnosis to MRD assessment. This links persistence of organ dysfunction and damage with permanence of even minimal clonal disease, producing undetectable, but still toxic amounts of LCs. This observation further corroborates other clinical and laboratory data, indicating a toxic effect of the circulating amyloid precursor^30–33^. Thus, efforts to improve the rate of organ response should aim at deepening hematologic response, possibly eradicating the PC clone. As such, high-sensitive NGF becomes a clinically relevant biomarker to confirm if the achievement of aCR is associated with profound eradication of clonal PCs, and to monitor the reappearance of MRD before hematological relapse as potential surrogate of upcoming organ dysfunction.

Undetectable MRD was also associated with longer progression-free survival. About one quarter of patients with persistent MRD experienced a hematologic progression (loss of aCR), while only one patient progressed among the 42 patients with MRD negativity. Of note, the sensitivity achieved by NGF for this single patient progressing was 10^−5^, further stressing the importance of reaching the highest sensitivity threshold with current MRD methodologies (i.e., 2 × 10^−6^ for NGF and 1 × 10^−6^ for NGS).

In conclusion, NGF can detect MRD in patients with AL amyloidosis otherwise in aCR, and persistent MRD can explain persistent organ dysfunction and predict/anticipate hematologic progression. Testing for MRD should be offered to subjects who attain aCR, especially if aCR is not accompanied by organ response. In case MRD is present, further chemotherapy could be considered, carefully balancing residual organ damage, patient frailty, and possible toxicity.

## Supplementary information

Supplemental material
