# Supplementary material for: Minimal residual disease negativity by next-generation flow cytometry is associated with improved organ response in AL amyloidosis
Source: Blood Cancer J. 2021 Feb 16;11(2):34. doi: 10.1038/s41408-021-00428-0 (PMC7887224; doi:10.1038/s41408-021-00428-0)
Supplement: Supplementary file 1 — Supplemental material [file 41408_2021_428_MOESM1_ESM.pdf]

Supplementary Material

Minimal residual disease by next-generation flow cytometry hinders organ response in AL amyloidosis patients

Giovanni Palladini,[1, 2, 3] Bruno Paiva,[4] Ashutosh Wechalekar,[5] Margherita Massa,[2] Paolo Milani,[1, 2] Marta Lasa,[4] Marco Basset,[1, 2, 3] Leire Burgos,[4] Mario Nuvolone,[1, 2, 3] Ramón Lecumberri,[4] Andrea Foli,[1, 2] Noemi Puig,[6] Melania Sesta,[1, 2, 3] Margherita Bozzola,[1, 2, 3] Pasquale Cascino,[1, 2, 3] Alice Nevone,[1, 2, 3] Jessica Ripepi, [1, 2, 3] Pierpaolo Berti,[7] Simona Casarini,[1, 2] Ombretta Annibali,[7] Alberto Orfao,[6] Jesus San Miguel,[4] Giampaolo Merlini[1, 2, 3]

[1]Amyloidosis Research and Treatment Center, "Fondazione Istituto di Ricovero e Cura a Carattere Scientifico (IRCCS) Policlinico San Matteo", Pavia, Italy

[2]Biochemistry, Biotechnology and Advanced Diagnostics Laboratory, "Fondazione Istituto di Ricovero e Cura a Carattere Scientifico (IRCCS) Policlinico San Matteo", Pavia, Italy

[3]Department of Molecular Medicine, University of Pavia, Pavia, Italy

[4]Clinica Universidad de Navarra, Centro de Investigacion Medica Aplicada (CIMA), IDISNA, CIBERONC Pamplona, Pamplona, Spain

[5]National Amyloidosis Centre, University College London Medical School, United Kingdom

[6]Servicio General de Citometría, Universidad de Salamanca, IBSAL, and IBMCC CSIC-USAL, CIBERONC, Salamanca, Spain

[7]Hematology and Stem Cell Transplantation Unit, University Campus Bio-medico, Rome, Italy.

Corresponding author:

Prof. Giovanni Palladini, MD, PhD

Amyloidosis Research and Treatment Center

Fondazione IRCCS Policlinico San Matteo

Viale Golgi, 19 – 27100 Pavia, Italy

telephone: +39-0382-502994

fax: +39-0382-502990

e-mail: giovanni.palladini@unipv.it

Supplementary Table 1.

Center	Number (%) of patients evaluated
Pavia Amyloidosis Center (Italy)	45 (49)
National Amyloidosis Center (London)	15 (17)
Hospital Universitario Puerta del Hierro (Spain)	11 (12)
Salamanca Hospital (Spain)	4 (4)
Hospital Universitario Dr Peset (Spain)	4 (4)
Hospital Universitario Son Espases (Spain)	2 (2)
Hospital de Cabuenes (Spain)	2 (2)
Hospital de Galdakao (Spain)	2 (2)
Hospital Son Llatzer (Spain)	2 (2)
Clinical Universidad de Navarra (Spain)	1 (1)
Complejo Hospitalario de Ourense (Spain)	1 (1)
Burgos Hospital (Spain)	1 (1)
Costa del Sol Hospital (Spain)	1 (1)
Valladolid Hospital (Spain)	1 (1)

Supplementary Table 2. Logistic regression for organ response.

**Variable**	**OR (95% CI)**	**P**
**Time to MRD assessment from aCR**		
Cardiac response	1.03 (0.96, 1.10)	P=0.157
Renal response	0.99 (0.97, 1.01)	P=0.653
**Time to aCR assessment from diagnosis**		
Cardiac response	0.97 (0.86, 1.08)	P=0.154
Renal response	0.95 (0.84, 1.08)	P=0.420

aCR, amyloid complete response; CI, confidence interval; MRD, minimal residual disease; OR, odds ratio.
